# Understanding headache classification coding within the veterans health administration using ICD-9-CM and ICD-10-CM in fiscal years 2014–2017

**DOI:** 10.1371/journal.pone.0279163

**Published:** 2023-01-04

**Authors:** Samah Jamal Fodeh, Brenda T. Fenton, Rixin Wang, Melissa Skanderson, Hamada Altalib, Deena Kuruvilla, Emmanuelle Schindler, Sally Haskell, Cynthia Brandt, Jason J. Sico

**Affiliations:** 1 Veterans Health Administration Headache Centers of Excellence Research and Evaluation Center, VA Connecticut Healthcare System, West Haven, CT, United States of America; 2 Pain Research, Informatics, and Multi-morbidities, and Education (PRIME) Center, VA Connecticut Healthcare System, West Haven, CT, United States of America; 3 Yale School of Public Health, New Haven, CT, United States of America; 4 Yale Center for Medical Informatics, New Haven, CT, United States of America; 5 Department of Emergency Medicine, Yale School of Medicine, New Haven, CT, United States of America; 6 Neurology Service, VA Connecticut Healthcare System, West Haven, CT, United States of America; 7 Clinical Epidemiology Research Center (CERC), VA Connecticut Healthcare System, West Haven, CT, United States of America; 8 Department of Neurology, Yale School of Medicine, New Haven, CT, United States of America; 9 Center for NeuroEpidemiological and Clinical Neurological Research, Yale School of Medicine, New Haven, CT, United States of America; 10 Primary Care Service, VA Connecticut Healthcare System, West Haven, CT, United States of America; 11 Comprehensive Women’s Health, VA Central Office, Chester, VA, United States of America; 12 Department of Internal Medicine, Yale School of Medicine, New Haven, CT, United States of America; Tabriz University of Medical Sciences, ISLAMIC REPUBLIC OF IRAN

## Abstract

**Objectives:**

Understand the continuity and changes in headache not-otherwise-specified (NOS), migraine, and post-traumatic headache (PTH) diagnoses after the transition from ICD-9-CM to ICD-10-CM in the Veterans Health Administration (VHA).

**Background:**

Headache is one of the most commonly diagnosed chronic conditions managed within primary and specialty care clinics. The VHA transitioned from ICD-9-CM to ICD-10-CM on October-1-2015. The effect transitioning on coding of specific headache diagnoses is unknown. Accuracy of headache diagnosis is important since different headache types respond to different treatments.

**Methods:**

We mapped headache diagnoses from ICD-9-CM (FY 2014/2015) onto ICD-10-CM (FY 2016/2017) and computed coding proportions two years before/after the transition in VHA. We used queries to determine the change in transition pathways. We report the odds of ICD-10-CM coding associated with ICD-9-CM controlling for provider type, and patient age, sex, and race/ethnicity.

**Results:**

Only 37%, 58% and 34% of patients with ICD-9-CM coding of NOS, migraine, and PTH respectively had an ICD-10-CM headache diagnosis. Of those with an ICD-10-CM diagnosis, 73–79% had a single headache diagnosis. The odds ratios for receiving the same code in both ICD-9-CM and ICD-10-CM after adjustment for ICD-9-CM and ICD-10-CM headache comorbidities and sociodemographic factors were high (range 6–26) and statistically significant. Specifically, 75% of patients with headache NOS had received one headache diagnoses (Adjusted headache NOS-ICD-9-CM OR for headache NOS-ICD-10-CM = 6.1, 95% CI 5.89–6.32. 79% of migraineurs had one headache diagnoses, mostly migraine (Adjusted migraine-ICD-9-CM OR for migraine-ICD-10-CM = 26.43, 95% CI 25.51–27.38). The same held true for PTH (Adjusted PTH-ICD-9-CM OR for PTH-ICD-10-CM = 22.92, 95% CI: 18.97–27.68). These strong associations remained after adjustment for specialist care in ICD-10-CM follow-up period.

**Discussion:**

The majority of people with ICD-9-CM headache diagnoses did not have an ICD-10-CM headache diagnosis. However, a given diagnosis in ICD-9-CM by a primary care provider (PCP) was significantly predictive of its assignment in ICD-10-CM as was seeing either a neurologist or physiatrist (compared to a generalist) for an ICD-10-CM headache diagnosis.

**Conclusion:**

When a veteran had a specific diagnosis in ICD-9-CM, the odds of being coded with the same diagnosis in ICD-10-CM were significantly higher. Specialist visit during the ICD-10-CM period was independently associated with all three ICD-10-CM headaches.

## Introduction

Headache is one of the most commonly diagnosed chronic conditions managed within primary and specialty care clinics [1, 2]. Headache has a lifetime prevalence of 66% [2–9]. Half of people with a headache history actively experience headache attacks [8, 10]. Understanding the public health impact of headache and the treatment needs of people with headache requires accurate data, especially when reporting disease prevalence. The Veterans Health Administration (VHA) is the largest integrated healthcare system within the United States (U.S.) and provides electronic health record (EHR) data over time for veteran enrollees [11]. The VHA transitioned from the International Classification of Diseases and Related Health Problems, 9^th^ revision, Clinical Modification (ICD-9-CM) to the 10^th^ revision on October 1, 2015. From this point onward, each ICD-9-CM diagnosis required manual recoding as an ICD-10-CM diagnosis in the EHR by healthcare provides managing headache.

The new ICD-10-CM codes were expected to increase diagnostic specificity, hence aiding healthcare providers, payers, and policymakers in understanding disease prevalence, establishing appropriate reimbursement rates, and improving care quality and delivery. While resources exist to map ICD-9-CM conditions onto ICD-10-CM, [12,13] the effect of this transition on coding of specific headache diagnoses is unknown. Accuracy of headache diagnosis is important since different headache types respond to different treatments, and newer, more expensive migraine-specific treatments, are emerging rapidly and may not be available to patients unless they are accurately diagnosed and coded as having migraine.

While coding of healthcare conditions such is guided by the ICD, diagnosis of headache conditions is guided by the International Classification of Headache Disorders (ICHD). This classification schema initially divides headache diseases into primary (e.g., migraine) and secondary (e.g., post-traumatic headache [PTH] headache. Primary headaches are those for which there is no known underlying etiology, whereas secondary headaches are attributable to an underlying condition or conditions thought to cause or have close temporal relationship to when a headache condition began [14, 15]. The first edition of the ICHD criteria was published in 1988 whereas its fourth edition is currently being developed [15, 16]. Headache classification and criteria have evolved over time, incorporating medicine’s growing understanding of headache conditions and the utility of previously used criteria in guiding clinicians in making distinct headache diagnoses [15, 17]. For example, when considering patients with migraine with typical aura, the ICHD-3 beta diagnostic criteria had a false positive rate of 16.9%, whereas its predecessor, the ICHD-3, had a false positive rate of 10.5%, as compared to the diagnosis made by treating physicians [17]. It should also be noted that, while there is no diagnostic category in ICHD for “headache,” ICD does contain a “headache” symptom code.

Clinicians were using the ICHD-3 beta criteria to diagnose headache diseases during the time when the VHA transitioned from ICD-9-CM to ICD-10-CM, clinicians were using the ICHD-3 beta criteria, which was introduced in 2013 and supplanted by ICHD-3 in 2018. Previous work mapping any headache diagnosis with the transition from ICD-9-CM to ICD-10-CM noted a 0.75 odds ratio of being coded with a headache disorder in ICD-10-CM if an ICD-9-CM code existed for any type of headache [3, 18]. Little if any work has examined diagnostic trajectories of specific headache conditions with the transition from ICD-9-CM to ICD-10-CM. After mapping ICD-9-CM headache diagnoses onto the ICD-10-CM system, we sought to understand the continuity and specificity of coding specific headache subtypes. We also explored whether provider type (primary care versus specialty care) and/or patient characteristics (age, sex, race/ethnicity) influenced ICD-10-CM coding.

## Materials and methods

### Study design

We measured headache coding proportions two years before and two years after the transition to ICD-10-CM to examine changes in headache classifications unadjusted by patient or characteristics. All patients included in the analyses had at least one VHA visit during the first two years of ICD-10-CM utilization (FY2016/2017) to allow for an opportunity for a headache condition to be coded. We utilized eight categories of headache present in both ICD-9-CM and ICD-10-CM coding systems: migraine, tension-type headache, trigeminal-autonomic-cephalalgias (TAC), other primary headache disorders, PTH, post-whiplash headache, other secondary headache disorders, and headache NOS.

### Mapping headache diagnoses from ICD-9-CM onto ICD-10-CM

The ICD-10-CM is a system used by healthcare providers to classify and code all diagnoses, symptoms and procedures recorded in conjunction with health care in the US. An ICD-10-CM is a seven-character, alphanumeric code. Each code begins with a letter, and that letter is followed by two numbers. The first three characters of ICD-10-CM are the “category.” The category describes the general type of the injury or disease. The category is followed by a decimal point and the subcategory. For example, G43 is “migraine,” whereas G43.1XX is “migraine with aura.” To develop corresponding ICD-10-CM diagnoses for the headache conditions above, we cross-walked the original ICD-9-CM codes for each condition coded in in FY 2014–2017 to ICD-10-CM codes using general equivalency mapping (GEMs). As such, the period in this study occurred exclusively during a time after ICHD-3 beta was published and before ICHD-3 was published (i.e., 2013 to 2018). Publicly available codes from the Centers for Disease Control and the Centers for Medicare and Medicaid Services [12] were used and cross-referenced with guidance from the American Academy of Neurology (AAN) [19] and the American Headache Society (AHS) [20]. The code mappings in Table 1 were independently reviewed by four neurologists specializing in headache care–two United Council for Neurologic Subspecialties (UCNS) Headache Medicine Certified and two UCNS Headache Medicine eligible neurologists. This crosswalk was applied to the Women Veterans Cohort Study (WVCS) of 1.14 million OEF/OIF/OND veterans with and without headache in order to update the WVCS cohort with the full complement of headache diagnoses to be examined in the VHA Headache Centers of Excellence (HCoE) cohort [11].

**Table 1:** ICD-9-CM/ICD-10-CM diagnostic codes for headache crosswalk.

Headache Type	ICD-9-CM	ICD-10-CM
Headache, NOS	784	R51.
Migraine	346.XX	G43.XXX, G43.BX
Migraine, without aura	346.1X	G43.0XX
Migraine, with aura	346.0X	G43.1XX
Chronic Migraine	346.7X	G43.7XX
Tension Headache	307.81, 339.1X	G44.2XX
TAC	339.0X, 339.41	G44.0XX, G44.51
Cluster Headache	339.00, 339.01, 339.02	G44.00X, G44.01X, G44.02X
Hemicrania	339.03, 339.04, 339.41	G44.03X, G44.04X, G44.51
Other Primary Headache Disorders	339.42, 339.43, 339.44, 339.8X	G43.CX, G44.52, G44.53, G44.54, G44.59, G44.8X
Post-Traumatic Headache	339.2X	G44.3XX
Post-Whiplash Headache	847	S13.4XXX, S13.8XXX, S13.9XXX
Other Secondary Headache Disorders	339.3	G44.1, G44.4X
Vascular Headache	-	G44.1
Drug-Induced Headache	339.3	G44.4X

Abbreviations: International Classification of Diseases (ICD); Clinical Modification (CM); Not-otherwise-specified (NOS); trigeminal autonomic cephalalgia (TAC).

### Specificity and utilization analysis of ICD-10-CM headache coding

We used structured queries to determine the ICD-9-CM to ICD-10-CM pathway at the VHA focusing on three headache diagnoses: Headache NOS (ICD-9-CM code = 784.XX; ICD-10-CM code = R51), migraine (ICD-9-CM code = 346.XX; ICD-10-CM code = G43.XXX, G43.BX), and PTH (ICD-9-CM code = 339.2X; ICD-10-CM code = G44.3XX). Thus, we retrieved veterans who have been coded for these headache types during the last two FYs of ICD-9-CM utilization (i.e., FY2014 or FY2015). We followed the coding of these same individuals in the first two fiscal years of ICD-10-CM (i.e., FY2016 and FY2017). For each headache type, our analysis was three-fold. First, we investigated the specificity and continuity of coding. Patients were identified as (1) no headache coded at all (e.g., headache NOS in ICD-9-CM and no headache diagnosis in ICD-10-CM); (2) the same headache disorder coded (e.g., headache NOS in both ICD-9-CM and ICD-10-CM); or (3) a new headache diagnosis or more than one new headache diagnoses coded instead of the original headache type (e.g., headache NOS in ICD-9-CM followed by migraine in ICD-10-CM). Second, we investigated changes in ICD-9-CM/ICD-10-CM coding relative to age, sex, and race/ethnicity. Third, we examined ICD-10-CM coding by different types of providers, comparing utilization by generalists (i.e., primary care, emergency department and urgent care) and specialists (i.e., neurologists and physiatrists). Provider type was based on stop codes that indicate clinic type as shown in S1 Table.

### Multivariate modeling of ICD-10-CM headache coding

Multivariate modeling was used to quantify the likelihood of receiving a given diagnosis of headache NOS, migraine, or PTH in ICD-10-CM when the original ICD-9-CM diagnosis of headache NOS, migraine, or PTH was made by a PCP, controlling for: ICD-9-CM headache diagnoses, ICD-10-CM headache diagnoses, and sociodemographic factors. Persons diagnosed in ICD-9-CM by other provider types were not included in these analyses, given that: (1) primary care providers manage a majority of patients with headache, and; (2) we sought to understand whether headache coding changed when a specialist was involved after the transition to ICD-10-CM. Adjusted odds ratios (aORs) and associated 95% Wald confidence intervals (CIs) were generated using logistic regression models containing the following variables: headache NOS, migraine, and PTH in ICD-9-CM and ICD-10-CM, followed by patient age, sex, and race/ethnicity.

Logistic regression was used to determine the odds of a specific headache diagnosis in ICD-10-CM being associated with a specific ICD-9-CM headache diagnosis made by a primary care provider, controlling for other headache morbidity, ICD-10-CM diagnoses made by either a generalist or specialist, age, sex, and race/ethnicity. A three-level provider type variable for the ICD-10-CM visit(s) was created. Generalists consisted of visits to either primary care, urgent care, or emergency care whereas specialists were classified as either neurologists or physiatrists. Diagnostic codes for neurology visits (6.74%) were coded first, regardless of whoever else saw the veteran. Physiatry clinic visits (3.51%) were coded next and were assigned if the patient was not seen in a neurology clinic and regardless if they were also seen by a generalist. The physician of record would be a generalist only if the patient was not seen by either neurology or physiatry services (89.75%). SQL server version 2014 was used to run queries and Python version 3.7.4 were used to generate Figures. Logistic regression was performed using SAS, version 9.4 (SAS Institute, Cary, NC).

## Results

**Fig 1:**
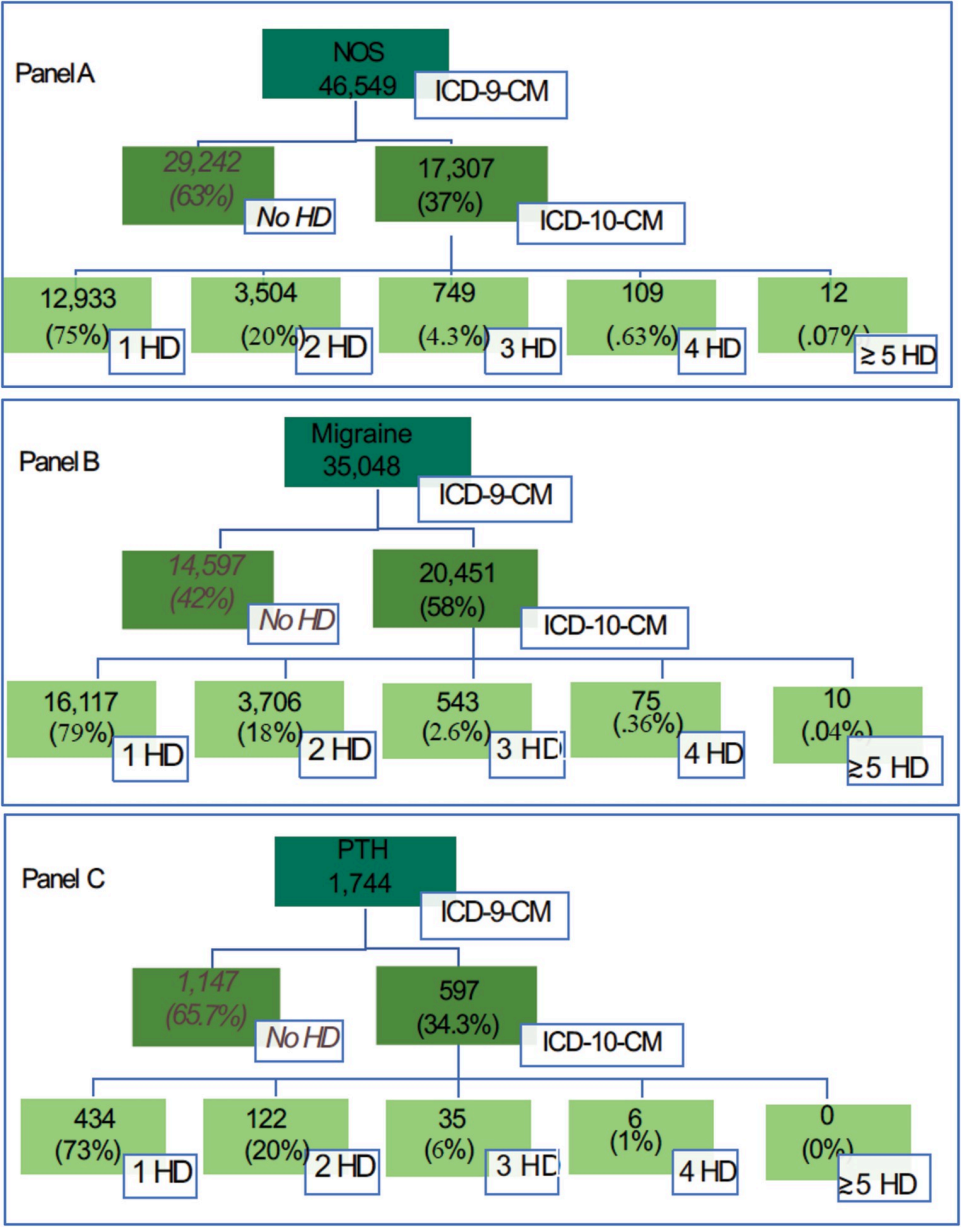
Distribution of patients based on number of headache diagnoses in ICD-10-CM. Abbreviations: Not-otherwise-specified (NOS); International Classification of Diseases, 10^th^ edition, Clinical Modification (ICD-10-CM); Headache Diagnoses (HD); Post-Traumatic Headache (PTH).

We report herein on the presence/absence of headache diagnoses as well as specific diagnostic patterns in ICD-10-CM. Fig 1 shows the breakdown of the number of ICD-10-CM headache diagnoses received by their ICD-9-CM headache diagnosis of interest (NOS-Panel A, migraine-Panel B or PTH-Panel C).

Table 2 then details the ICD-10-CM diagnoses in FY 2016/2017 for patients coming out of FY 2014/2015 with ICD-9-CM coding for NOS, migraine and PTH in the the Panels A, B, C, respectively. The rows and the columns in the table encode all possible ICD-10-CM diagnoses that existed in FY2016/2017. The cell son the diagonals (bolded) show the number of patients who received one headache diagnosis, whereas the off-diagonal cells define the number of patients who received a combined diagnosis of two different headache types.

**Table 2:** Specificity of ICD-10-CM headache coding among patients who were diagnosed with headache diagnosis of NOS, migraine, or PTH based on ICD-9-CM.

Panel A—Headache NOS Coding in ICD-10-CM								
Headache Coding (ICD-10-CM)	NOS	Other Primary	PTH	Other Secondary	TAC	Tension	Migraine	Whiplash
**NOS**	**8556**							
**Other Primary**	184	**218**						
**PTH**	242	9	**347**					
**Secondary**	42	4	2	**51**				
**TAC**	51	3	1	1	**68**			
**Tension**	355	13	9	3	2	**448**		
**Migraine**	2168	51	112	35	17	137	**3152**	
**Whiplash**	45	1	2	0	0	0	15	**93**
**Panel B- Migraine Coding in ICD-10-CM**								
**NOS**	**1945**							
**Other Primary**	38	**72**						
**PTH**	49	3	**86**					
**Secondary**	10	1	1	**51**				
**TAC**	12	1	0	0	**27**			
**Tension**	41	2	3	0	0	**106**		
**Migraine**	2895	115	138	74	29	225	**13788**	
**Whiplash**	16	0	2	0	0	0	51	**42**
**Panel C—PTH Coding in ICD-10-CM**								
**NOS**	**156**							
**Other Primary**	5	**7**						
**PTH**	31	1	**135**					
**Secondary**	0	0	0	**4**				
**TAC**	0	0	0	0	**1**			
**Tension**	7	0	0	0	0	**12**		
**Migraine**	44	1	25	1	0	4	**116**	
**Whiplash**	2	0	0	0	0	0	1	**3**

**Abbreviations:** International Classification of Diseases, 10^th^ edition, Clinical Modification (ICD-10-CM); Not-otherwise-specified (NOS); Post-Traumatic Headache (PTH); Trigeminal autonomic cephalalgia (TAC); Bolded diagonals indicate only one headache diagnosis.

### Headache diagnoses & specifics in ICD-10-CM (FY2016-FY2017)

#### Headache NOS

**Fig 2:**
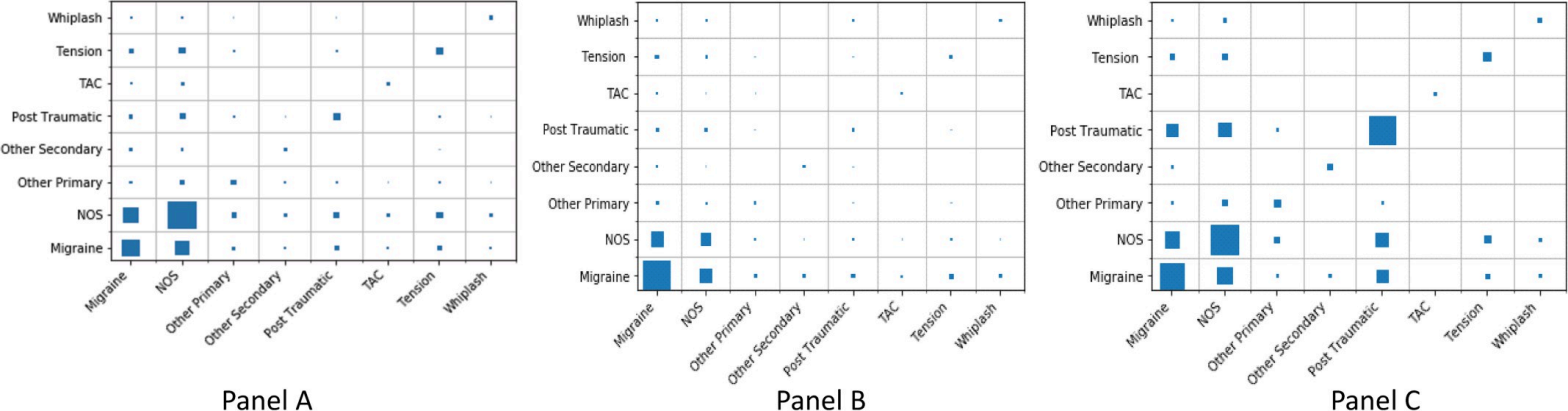
Panel-A: Headache NOS coding. Panel-B: Migraine headache coding. Panel-C: PTH headache coding in FY2016/2017. Abbreviations: Not-otherwise-specified (NOS); Trigeminal autonomic cephalalgia (TAC); Post-traumatic headache (PTH).

In considering the pathways of headache from FY2014/2015 into FY 2016/2017 for patients with an ICD-9-CM diagnosis of headache NOS (Fig 1, Panel A), 63.2% (29,424/46,549) of patients did not receive any ICD-10-CM headache diagnostic code. Of the headache NOS patients (N = 17,307) who were coded with an ICD-10-CM headache diagnosis in FY2016 and FY2017, 75% (N = 12,933/17,307) had one headache diagnosis within any of the eight headache categories; two-thirds of these patients (66%, 8556/12,993) were recoded as headache NOS in ICD-10-CM, while the remaining patients received a new, different headache diagnosis (Table 2, Panel A). About 20% (3,504/17,307) of patients had two headache diagnoses with the remaining 5% (870/17,307) receiving three or more distinct headache codes (Fig 1, Panle A). The prevalence of ICD-10-CM diagnoses for patients with ICD-9-CM coded headache NOS were: headache NOS (49%; 8,556/17,307), migraine (18%; 3,152/17,307), and tension-type headache (3%; 448/17,307; see Table 2 Panel A). In considering multiple diagnoses, approximately 13% (2,168/17,307) of patients carried the combination of headache NOS and migraine diagnoses compared to 2% (355/17,307) of patients who were coded as the combination of headache NOS and tension-type headache. Few patients with headache NOS received multiple specific headache diagnoses, including those receiving the combination of migraine and tension-type headache (0.8%; 137/17,307) and migraine and PTH (0.6%; 112/17,307). Fig 2 with Panels A, B, C shows a visual representation of the corresponding panels in Table 2. Both axes in a given panel refer to ICD-10-CM coding as this representation visualizes the details of ICD-10-CM coding in FY 2016/2017. However, each panel shows coding for patients with a certain headache based on ICD-9-CM in FY 2014/2015. For example, Panel A, shows the details of the ICD-10-CM coding for patients who were coded for NOS headache based on ICD-9-CM in FY 2014/2015. The larger the size of the square within a cell, the greater number of patients that fall in that cell.

#### Migraine headache

In considering the pathways of headache from FY2014/2015 into FY 2016/2017 for patients with an ICD-9-CM migraine headache diagnosis (Fig 1, Panel B), 41.6% (14,597/35,048) of patients did not receive any ICD-10-CM headache diagnostic code. As shown in Fig 1 (Panel B), about 79% (16,117/20,451) of coded patients in the first two years of ICD-10-CM implementation received a single headache diagnosis. Among these patients, 86% (13,788/16,117) retained the same single diagnosis coded in ICD-9-CM whereas the remaining 14% (2,329/16,117) received a new headache diagnosis in ICD-10-CM (Table 2 –Panel B); Ten percent (1,945/20,451) of all patients coded with migraine in ICD-9-CM received the less specific diagnosis of headache NOS in ICD-10-CM. A very small percent of cases switched from migraine to a single headache diagnosis of another type: approximately 0.5% (106//20,451), 0.4% (86/20,451), and 0.3% (72/20,451) of migraine patients received a single diagnosis of tension-type headache, PTH, and other primary headache disorder, respectively rather than being diagnosed with migraine in ICD-10-CM (Table 2, Panel B). Eighteen percent (3,706/20,451) of patients (with only the diagnosis of migraine in ICD-9-CM) received two headache diagnoses in ICD-10-CM (Fig 1, Panel B), with 78% (2,895/3706) of these patients being coded with the combination of both migraine and headache NOS (Table 2, Panel B). Three percent (628/20,451) of patients received more than two headache diagnoses (Fig 1, Panel B).

#### Post-Traumatic Headache (PTH)

In considering the pathways of headache from FY2014/2015 into FY 2016/2017 for patients with an ICD-9-CM PTH diagnosis (Fig 1, Panel C), 65.8% (1,147/1,744) of patients did not receive any ICD-10-CM headache diagnostic code. Of those assigned at least one ICD-10-CM headache diagnostic code, 73% (434/597) had a single headache diagnosis after the ICD-9-CM/ICD-10-CM transition. As shown in (Table 2, Panel C), 23% (135/597) of PTH patients continued to be coded as only PTH, whereas 26% (156/597) and 19% (116/597) were coded with headache NOS and migraine only, respectively. Twenty percent (122/597) of patients with only the ICD-9-CM diagnosis of PTH received two headache diagnoses in ICD-10-CM (Fig 1, Panel C), with headache NOS and migraine diagnoses added to the PTH code for about 5% (31/597) and 4% (25/597) patients, respectively (Table 2, Panel C). Approximately 7% (44/597) of PTH patients were newly coded with migraine and headache NOS after ICD-10-CM implementation, while near seven percent of patients (41/597) had more than two headache diagnoses (Fig 1, Panel C).

### Utilization of ICD-10-CM headache coding per provider type

**Fig 3:**
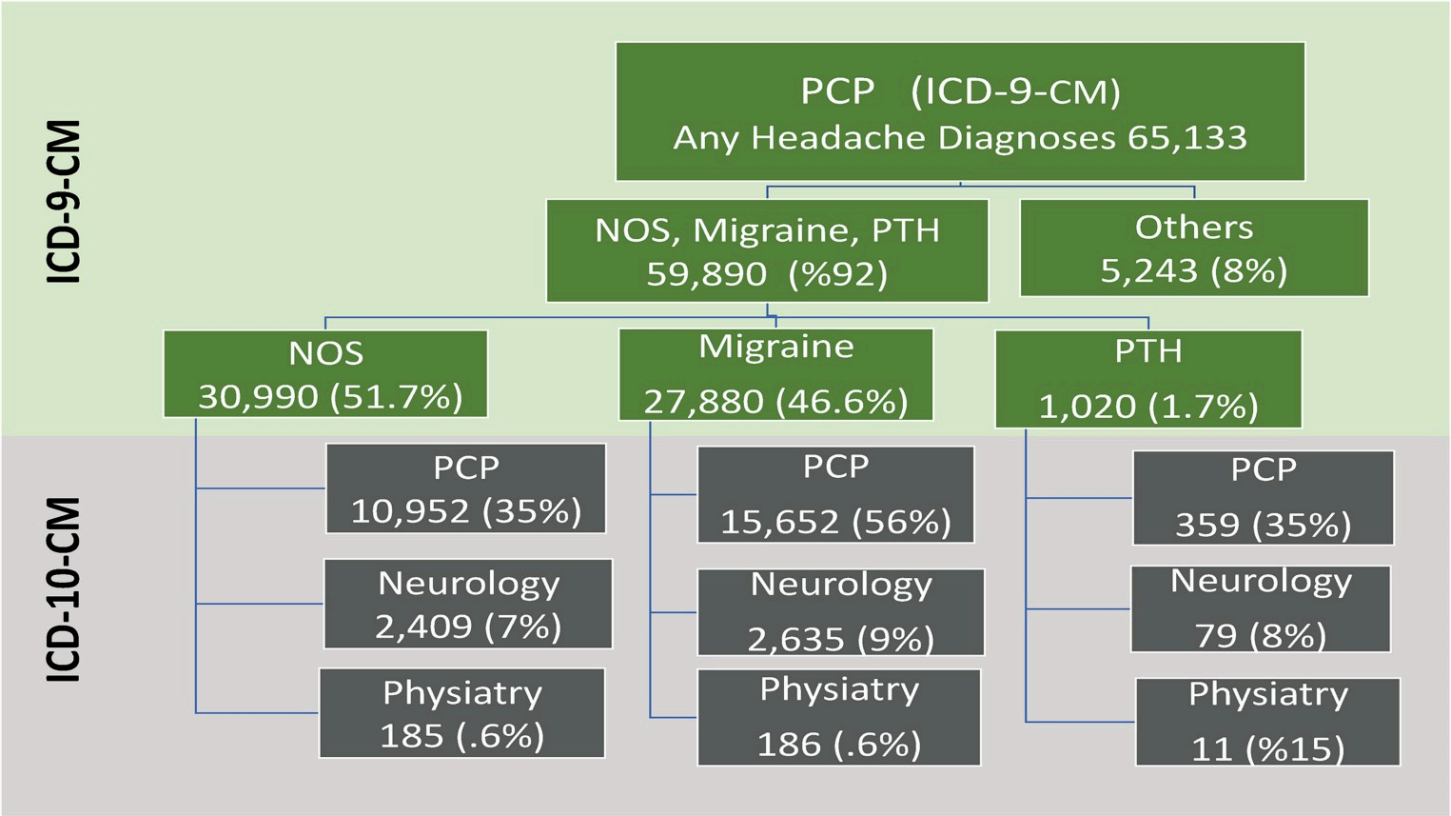
Utilization of ICD-10-CM headache coding per provider type as shown in the black colored boxes.

A total of 65,133 patients were seen exclusively by PCPs and coded with any of the eight headaches diagnosis in FY2014-FY2015. Of those, 59,890 patients were diagnosed with NOS, migraine, or PTH. Roughly half of the patients (51.7%, 30,990/59,890) were coded for NOS headache, 46.6% (27,880/59,890) with migraine, and 1.7% (1,020/59,890) with PTH. In FY2016-FY2017, patients with headache NOS seen only by a PCP in FY2015-FY2016, 35% (10,952/30,990), 7.0% (2,409/30,990), and 0.6% (185/30,990) saw primary care only, neurology, and physiatry, respectively. Among patients with migraine seen only by a PCP during the last two years of ICD-9-CM, 56% (15,652/27,880), 9% (2635/27,880), and 0.6% (186/27,880) were seen by primary care only, neurology, and physiatry, respectively in the first two years of ICD-10-CM coding. Of patients with PTH seen by a PCP before the ICD transition, 35% (359/1,020), 8.0% (79/1,020), 1.0% (11/1,020) saw primary care only, neurology, and physiatry, respectively, after the transition as shown in Fig 3.

### Multivariate modeling of ICD-10-CM headache coding

Table 3 reports the adjusted odds ratios (aORs) and their 95% confidence intervals for ICD-10-CM headache NOS (as diagnosed by any provider type) with the original ICD-9-CM headache diagnosis by a PCP.

**Table 3:** Probability modeled is ICD-10-CM headache NOS, migraine, and post-traumatic headache, with and without modeling specialist/generalist.

Headache	Headache NOS		Migraine		PTH	
	Without Specialty	With Specialty	Without Specialty	With Specialty	Without Specialty	With Specialty
Characteristic	aOR (95% CI)					
*Coded Headache Diagnoses*						
Headache NOS ICD-9-CM (PCP)	6.10	5.71	2.33	2.28	2.74	2.66
	(5.89, 6.32)	(5.50, 5.92)	(2.23, 2.44)	(2.18, 2.40)	(2.44, 3.10)	(2.36, 3.00)
Migraine ICD-9-CM (PCP)	1.20	1.30	26.43	27.87	1.00	1.10
	(1.14, 1.27)	(1.23, 1.37)	(25.51,27.38)	(26.83,28.95)	(0.85, 1.17)	(0.93, 1.28)
PTH ICD-9-CM (PCP)	1.27	1.28	1.47	1.46	22.92	22.47
	(1.07, 1.51)	(1.07, 1.53)	(1.22, 1.77)	(1.21, 1.78)	(18.97, 27.68)	(18.51,27.29)
Headache NOS ICD-10-CM			4.36	2.37	6.11	3.36
			(4.17, 4.57)	(2.26, 2.49)	(5.44, 6.87)	(2.98, 3.79)
Migraine ICD-10-CM	4.15	2.26			3.79	1.98
	(3.96, 4.34)	(2.15, 2.36)			(3.31, 4.32)	(1.73, 2.26)
PTH ICD-10-CM	5.94	3.19	3.98	2.07		
	(5.29, 6.68)	(2.83, 3.60)	(3.49, 4.55)	(1.80, 2.37)		
*Sociodemographic*						
Female	1.26	1.27	1.92	1.97	0.42	0.42
	(1.21, 1.31)	(1.21, 1.32)	(1.85, 2.00)	(1.88, 2.05)	(0.34, 0.51)	(0.34, 0.51)
Age	0.99	0.98	0.99	0.99	0.98	0.97
	(0.99, 0.99)	(0.98, 0.98)	(0.99, 1.00)	(0.98, 0.99)	(0.97, 0.98)	(0.96, 0.98)
Black versus White	1.29	1.23	1.21	1.16	0.66	0.65
	(1.24, 1.34)	(1.19, 1.28)	(1.16, 1.26)	(1.11, 1.22)	(0.56, 0.77)	(0.55, 0.76)
Hispanic versus White	1.31	1.23	1.09	1.02	0.88	0.82
	(1.25, 1.37)	(1.17, 1.28)	(1.03, 1.15)	(0.97, 1.08)	(0.75, 1.04)	(0.70, 0.97)
Other versus White	1.02	1.01	1.03	1.01	0.88	0.84
	(0.96, 1.09)	(0.94, 1.07)	(0.97, 1.10)	(0.94, 1.08)	(0.71, 1.10)	(0.68, 1.05)
*Generalist versus Specialist*						
Neurologist (v Generalist)		4.62		5.43		5.70
		(4.44, 4.81)		(5.18, 5.68)		(5.05, 6.43)
Physiatrist (v Generalist)		1.81		1.39		2.21
		(1.69, 1.94)		(1.27, 1.51)		(1.75, 2.79)

Abbreviations: Not-otherwise-specified (NOS); Post-Traumatic Headache (PTH); adjusted odds ratio (aOR); Confidence Intervals (CI); International Classification of Diseases, 9^th^ edition, Primary Care Provider (PCP)

The largest odds ratios are for the same diagnosis in ICD-9-CM:ICD-10-CM pairing (headache NOS aOR = 6.10, 95% CI: 5.89–6.32). An increased likelihood of headache NOS independently with both PCP-diagnosed migraine (aOR = 1.20, 95% CI: 1.14–1.27) and PTH diagnoses (aOR = 1.27, 95% CI: 1.07, 1.51) is seen as well. The aORs for ICD-10-CM headache diagnoses confirmed a significant amount of headache comorbidity (i.e., a different, comorbid headache diagnosis) between ICD-10-CM headache NOS and both ICD-10-CM migraine and PTH.

With respect to migraine headache in ICD-10-CM (Table 3), we see a similar pattern with the highest aORs for the migraine ICD-9-CM:ICD-10-CM pairing (migraine aOR = 26.43, 95% CI: 25.51–27.38). Significant crossover between headache types in ICD-9-CM and ICD-10-CM and significant headache comorbidity in ICD-10-CM were observed.

Lastly, the findings with respect to PTH are similar with respect to the high likelihood of the same headache being diagnosed across ICD-9-CM and 10 (Table 3, PTH aOR = 22.92, 95% CI: 18.97–27.68). In contrast to the other models, an ICD-9-CM migraine did not independently predict ICD-10-CM PTH (aOR = 1.00, 95% CI: 0.85–1.17), whereas ICD-10-CM migraine was highly comorbid with ICD-10-CM PTH (aOR = 3.79, 95% CI: 3.33–4.32). ICD-9-CM headache NOS remained a significant predictor of ICD-10-CM PTH (aOR = 2.74, 95% CI: 2.44–3.09). ICD-10-CM headache NOS was highly comorbid (aOR = 6.11, 95% CI: 5.44–6.87) with an ICD-10-CM PTH diagnosis. For all three headache types controlling for age, sex, and race/ethnicity only minimally reduced the odds ratio for ICD-9-CM to the same diagnoses in ICD-10-CM.

#### Changes in ICD-9-CM and ICD-10-CM headache coding across age, sex, and race/ethnicity

In Table 4 we present the changes in headache coding for the different sociodemographic factors. Headache NOS coding decreased by 12% among younger patients (i.e., 18–29 age group), while patients older than 30 years of age experienced an 11% increase in utilization of headache NOS coding. Coding of migraine decreased by 11% among younger patients and increased by a total of 9% for patients older than 30 years of age. A 20% increase in PTH was observed for younger patients whereas and a similar decrease occurred for patients older than 30 years of age. Females experienced a 2% increase in headache coding for headache NOS and PTH compared to a 2% decrease in males. Less headache NOS coding occurred among whites (2%) compared to a 1% increase in both Black and Hispanic populations. For migraine, patterns of coding did not change except for Hispanics, where an increase of 1% was observed in FY2016-FY2017. A 1% increase in PTH coding across all groups was observed except for Hispanics, who experienced a decrease of 3%.

**Table 4:** Changes in headache coding across different sociodemographic groups.

	ICD-9-CM						ICD-10-CM					
	NOS	%	MIG	%	PTH	%	NOS	%	MIG	%	PTH	%
Female	6646	14%	9891	28%	76	4%	5853	16%	10192	28%	101	6%
Male	39899	86%	25151	72%	1668	96%	30892	84%	26324	72%	1723	94%
Unknown	4	0.01%	6	0%	0	0.02%	3	0%	8	0%	0	0.02%
White	28217	60%	20687	59%	1226	70%	21402	58%	21561	59%	1295	71%
Black	9495	21%	7982	23%	211	12%	7867	22%	8336	23%	230	13%
Hispanic	6037	13%	3907	11%	222	13%	5191	14%	4202	12%	191	10%
Other	2844	6%	2503	7%	85	5%	2288	6%	2425	7%	108	6%
18–29	12025	26%	7328	21%	658	38%	5003	14%	4066	11%	332	18%
30–39	19887	43%	15680	45%	711	40%	18795	51%	18353	50%	983	54%
40–49	9773	21%	8599	25%	263	15%	7836	21%	9372	26%	343	19%
50–59	4253	9%	3126	9%	101	6%	4378	12%	4270	12%	148	8%
>60	611	1%	315	1%	11	1%	736	2%	463	1%	18	1%

Abbreviations: International Classification of Diseases, 10^th^ edition, Clinical Modification (ICD-10-CM); Not-otherwise-specified (NOS); Migraine (MIG); Post-Traumatic Headache (PTH).

Age, sex, and race/ethnicity were examined as independent factors for ICD-10-CM headache diagnoses in the models containing all 3 headache-ICD-9-CM diagnoses of interest, ICD-10-CM headache comorbidities and ICD-10-CM specialist visit. The odds of each of the ICD-10-CM headache diagnosis outcomes significantly decreased with increasing age. Women, (aOR = 1.26, 95% CI: 1.21–1.31) Black (aOR = 1.29, 95% CI: 1.24–1.34), and Hispanic veterans (aOR = 1.31, 95% CI: 1.25–1.37) were more likely to have an ICD-10-CM diagnosis of headache NOS after controlling for headache comorbidity, age, sex, and race/ethnicity. Adjusted for headache comorbidity and diagnostic crossover, the likelihood of being diagnosed with migraine was higher in women (aOR = 1.92, 95% CI: 1.85–2.00), Black (aOR = 1.21, 95% CI: 1.16–1.26), and Hispanic (aOR = 1.09, 95% CI: 1.03–1.15) veterans. In contrast to the findings above, women were less likely to be diagnosed with PTH (aOR = 0.42, 95% CI: 0.34–0.51) as were Black veterans (aOR = 0.66, 95% CI: 0.56–0.77).

#### Changes in ICD-9-CM and ICD-10-CM coding when specialists involved in headache diagnosis

In the first two years of ICD-10-CM implementation, relatively few people who had seen a PCP in the last two years of ICD-9-CM saw a specialist (10.25%). When considering the involvement of specialists in recording ICD-10-CM headache diagnoses, for predicting headache NOS in ICD-10-CM, all headache morbidity was significantly and positively associated with the probability of having headache NOS diagnosed. The largest odds ratio was for headache NOS in ICD-9-CM (aOR = 5.71, 95% CI: 5.50–5.92; Table 3). The type of provider seen in the ICD-10-CM two-year window was also significantly independently associated with the probability of a headache NOS diagnosis (Neurology [aOR = 4.62, 95% CI: 4.44–4.81] and Physiatry [aOR = 1.81, 95% CI: 1.69–1.94] compared to generalists. The same pattern emerged for migraine in ICD-10-CM; the largest odds ratio for migraine in ICD-9-CM (aOR = 27.87, 95% CI: 26.83–28.95) and ICD-10-CM visits with specialists resulted in higher odds of a migraine diagnosis (Neurology [aOR = 5.43, 95% CI: 5.18–5.68] and Physiatry [aOR = 1.39, 95% CI: 1.27–1.51]) than those where only a generalist was involved. Lastly, for ICD-10-CM PTH, we observed the same pattern for specific ICD-9-CM:ICD-10-CM headache types (PTH aOR = 22.47, 95% CI: 18.51–27.29) and the significance of specialists after controlling comorbidity in ICD-9-CM and ICD-10-CM (Neurology [aOR = 5.70, 95% CI: 5.05–6.43] and [Physiatry aOR = 2.21, 95% CI: 1.75–2.79]). In all three outcome models, the addition of the three-level specialist variable reduced the odds ratios for the other two headache comorbidities while remaining independently associated with the probability of diagnosis of each headache outcome. The addition of the specialist variables to the ICD-10-CM PTH model resulted in Hispanic veterans having significantly reduced odds of getting a PTH diagnosis.

## Discussion

Using the last two years of ICD-9-CM coding and first two years of ICD-10-CM coding in VHA, we mapped the same patients with an ICD-9-CM headache diagnosis onto the ICD-10-CM system. We also sought to understand the contribution of age, sex, race/ethnicity, and provider type in influencing headache coding during the earliest part of the transition to ICD-10-CM. In considering headache NOS, migraine, and PTH, several findings are noteworthy.

First, while patients received an ICD-9-CM diagnosis headache diagnosis, a majority of patients did not receive any ICD-10-CM code for headache despite having at least one office visit with a provider who could have coded an ICD-10-CM diagnosis within the EHR. Reasons for the lack of coding headache diagnoses in ICD-10-CM include: the headache condition may have resolved, the headache was stable and hence not an “active issue,” or may not have been the focus of the office visit. Not coding patients with headache diagnoses with the transition to ICD-10-CM has similarly been reported with VHA data, such that patients with any headache diagnosis prior to the transition had a 0.75 odds of receiving any headache diagnosis in the same ICD-10-CM time period examined here [21].

Second, when patients did receive an ICD-10-CM code, most patients continued receiving a single code which corresponded to their ICD-9-CM headache code. Among ICD-9-CM headache NOS patients, nearly half continued with a headache NOS during ICD-10-CM; 20% were coded as migraine in ICD-10-CM. When an additional diagnosis was given to ICD-10-CM headache NOS, it also tended to be migraine. As such, most patients with ICD-9-CM headache NOS diagnosis retained the least specific headache diagnosis possible or were either recoded as having a more specific headache diagnosis. More specific ICD-10-CM headache diagnosis may have resulted from a more complete headache evaluation during or after the transition of coding schemas, a “clean slate” of the patient’s health concerns (“problem list”) may have compelled the re-evaluation of headache history and diagnosis, and the new coding schema provided more diagnostic choices. Also, providers had additional time using ICHD-3 beta headache criteria in the first two years of the transition compared to the two prior years, which may also account for increased use of migraine diagnostic codes. Additional reasons for the recoding of ICD-9-CM headache NOS to ICD-10-CM migraine may be more intrinsic to the differences of these two-coding schema. For example, ICD-9-CM code of 346.20 (“variants of migraine, not elsewhere classified, without mention of intractable migraine without mention of status migrainosus”) has five corresponding ICD-10-CM codes, including G43.809 (“other migraine, not intractable, without status migrainosus”) and G43.D0 (“ophthalmoplegic migraine, not intractable”). However, for our analysis, we grouped all migraine ICD codes together; hence, the improved specificity of ICD-10-CM may be more applicable to individual types of migraine rather than migraine as a whole. Generalists and specialists both continued to use a headache NOS diagnosis, despite there being clear guidance in the International Classification of Headache Disorders 3^rd^ edition (ICHD-3) regarding the criteria for each headache type [9]. When ICD-9-CM diagnosed migraine patients received an ICD-10-CM code, it was overwhelmingly a code for migraine, either alone or in conjunction with a second code (most commonly headache NOS). Migraine may be the most familiar as well as the best understood of the headache disorders, given the high rates in which patients present to Primary and Specialty Care clinics for headache care [3, 7]. Another reason for the potential persistence of migraine diagnosis coding is that migraine is the only headache type allowed as a service-connected disability within VHA. ICD-9-CM diagnosed PTH showed the most changes in coding during ICD-10-CM with a greater percentage of these patients being coded with ICD-10-CM headache NOS and migraine or migraine alone. The migraine phenotype of PTH is the most commonly encountered, and may explain why these patients received an ICD-10-CM migraine diagnosis [22, 23].

Third, age, sex and race/ethnicity were independently associated with ICD-10-CM diagnoses adjusting for the three select headache diagnoses in both ICD-9-CM and ICD-10-CM. The decrease in all three headache types with increasing age and marked gender differences have been seen previously. The associations of race/ethnicity and headache coding across the three select headache diagnoses are interesting in the context of veterans being seen in the integrated healthcare system as well as adjusting for specialist visits. Black and Hispanic veterans were significantly less likely to receive an ICD-10-CM PTH diagnosis after adjusting for their ICD-9-CM diagnoses and ICD-10-CM comorbidity as well as age, sex, and specialist visits. These findings match what has been reported in the literature regarding disparities in headache care and may be explained by social constructs such as race and socioeconomic status [24].

Fourth, among those that did receive an ICD-10-CM code, only 10.25% saw a specialist for headache. In community-dwelling U.S. patients with at least one office-based visit, 10.93% of those with a self-reported history of headache saw a neurologist [25]. There is a marked shortage of neurologists and headache medicine providers to see patients with headache, especially within high-income countries [26, 27]. A similar shortage of neurologists, and particularly, headache neurologists, exists within the VHA. This has contributed to Congressional and VHA commitment to expanding access to headache medicine providers through the established of a national VHA Headache Centers of Excellence program.

Lastly, when patients saw providers with additional training and expertise in headache diagnosis and management, their odds of being diagnosed and coded with any of the three headache types of interest increased, compared to when only a generalist was involved. These findings highlight the importance of headache specialists in diagnosing and coding headache conditions; however, we did observe that even among specialists, patients continue to be coded with the least specific headache diagnosis. Adjustment in the models for specialist visits did not appreciably change the aORs for sociodemographic factors, suggesting these did not confound their observed associations with ICD-10-CM diagnoses.

Limitations to our work are worth noting. Although our study is a mapping study, it is considered a “transitioning study” due to its longitudinal study design (ICD-9-CM at FY 2014/2015 and ICD-10-CM at FY 2016/2017) and not cross-sectional study where both coding systems are used simultaneously. Therefore, the results are interpreted differently. Medically-diagnosed headache disorders and have not been validated by chart review or structured patient interviews. Next, it is possible that ICD-9-CM headache diagnoses were updated after the two-year period of observation that we selected. However, a longer period after the beginning of the transition could have allowed additional time for headaches to either change (e.g., to no longer become an “active issue”) or for patients to develop a new type of headache with or without an interceding event. Among patients who received two headache diagnoses in ICD-10-CM, we cannot comment if they developed a new headache disorder or if it was the same headache condition being coded differently. Again, keeping the examination period short hopefully minimized the appearance of new headache types in the interim between the ICD-9-CM and ICD-10-CM diagnoses. Next, coding may not be synonymous with diagnosis. Providers may have documented in the EHR a diagnosis of migraine and coded them as headache not otherwise specified. Formal chart review to determine the agreement between ICD-10-CM headache codes and unstructured clinic note data would be required to understand if this is a concern. Also, we considered a diagnosis by a specialist as superseding that of a generalist. While this hypothesis is supported by the additional headache training specialists receive, we are not aware studies comparing diagnostic and coding accuracy across provider types. Finally, whether greater degrees of headache severity, disability, or frequency may have impacted diagnoses which were coded was not explored, as these are not routinely available in administrative data.

## Conclusion

In the first two years where the largest integrated healthcare system in the U.S. transitioned to a coding schema intended to promote diagnostic specificity, we demonstrate both promise and opportunities for improvement and future inquiry. While headache NOS continued to be used commonly by generalists and specialists, there was a high rate of reclassification from headache NOS to another more specific headache diagnosis, chief among them being migraine, even after controlling for age, gender, and race/ethnicity. It is puzzling that patients with ICD-9-CM migraine or PTH diagnoses sometimes received a less specific diagnosis of headache NOS; this finding deserves further exploration to determine if there are any diagnostic challenges or systematic biases in the coding of this important headache diagnosis. Future work will also explore the impact of the ICD-9-CM/ICD-10-CM transition on other headache diagnoses as well as examined whether receipt of a more specific headache diagnosis or diagnoses changes healthcare utilization and headache care quality and delivery.

## Supporting information

S1 Table Clinical visits stop codes assigned to primary care providers, neurologists, and physiatrists. (DOCX)
