# Supplementary material for: Understanding headache classification coding within the veterans health administration using ICD-9-CM and ICD-10-CM in fiscal years 2014–2017
Source: PLoS One. 2023 Jan 4;18(1):e0279163. doi: 10.1371/journal.pone.0279163 (PMC9812322; doi:10.1371/journal.pone.0279163)
Supplement: S1 Table — (DOCX) [file pone.0279163.s001.docx]

Supplementary S1 Table: Clinical visits stop codes assigned to primary care providers, neurologists, and physiatrists

| **Provider Type** | **Visit Stop Code** | **Description** |
| --- | --- | --- |
| Primary Care Providers | 301 | Internal Medicine |
|  | 310 | Infectious Disease |
|  | 318 | Geriatrics |
|  | 322 | Women’s Primary Care |
|  | 323 | Primary Care |
|  | 324 | Telephone Medicine |
|  | 338 | Telephone Primary Care |
|  | 348 | Primary Care Group |
|  | 349 | Sleep Medicine |
|  | 404 | Gynecology |
|  | 704 | Women Gender-Specific Preventive Care |
| Neurologists | 315 | General Neurology |
|  | 212 | EMG |
| Physiatrists | 197 | Polytrauma |
|  | 201 | Physical Medicine & Rehab |
